# Supplementary material for: PI3k inhibitors (BKM120 and BYL719) as radiosensitizers for head and neck squamous cell carcinoma during radiotherapy
Source: PLoS One. 2021 Jan 20;16(1):e0245715. doi: 10.1371/journal.pone.0245715 (PMC7817006; doi:10.1371/journal.pone.0245715)
Supplement: Attachment — Submitted filename: response to reviewers_BKM_plos one.docx [file pone.0245715.s001.docx]

Review Comments to the Author

Please use the space provided to explain your answers to the questions above. You may also include additional comments for the author, including concerns about dual publication, research ethics, or publication ethics. (Please upload your review as an attachment if it exceeds 20,000 characters)

Reviewer #1: The authors present an interesting analysis of the use of PI3K inhibitors to sensitize OSCC models to radiation therapy that has some novelty in this field. At this point, the manuscript lacks substantial supporting experiments to warrant publication, and some recommendations to help the authors improve the manuscript are listed below.

1) Inclusion of pharmacodynamic analysis of PI3K effector signaling in the models presented, with the doses used (which are often very high).
**Our response:** As suggested by reviewer, we have considered the *in vitro* time-course pharmacodynamic analysis of the PI3K inhibitor, but we decided the assay will be performed in vivo study which may provide more information for the clinical trials. However, we still work on the development of the animal model because of the radiotherapy assay being not easy to perform.

2) Inclusion of pharmacodynamic analysis of DNA damage responses (e.g. gH2AX, COMET assays) following radiation, as well as more detailed analysis of mechanisms of cell death.
**Our response:** Previous study has demonstrated that BYL719 combined with RT induced the tumor γH2AX nuclear formation in HNSCC animal model (1). BKM120 also showed enhanced DNA damage activity in hepatocellular carcinoma cells (2). The detailed mechanisms we will perform in the HNSCC animal model in the further study.

**Reference**

1.	Mizrachi A, Shamay Y, Shah J, Brook S, Soong J, Rajasekhar VK, et al. Tumour-specific PI3K inhibition via nanoparticle-targeted delivery in head and neck squamous cell carcinoma. Nature communications. 2017;8:14292.

2.	Liu WL, Gao M, Tzen KY, Tsai CL, Hsu FM, Cheng AL, et al. Targeting Phosphatidylinositide3-Kinase/Akt pathway by BKM120 for radiosensitization in hepatocellular carcinoma. Oncotarget. 2014;5(11):3662-72.

3) In vivo modeling of the various combinations.
**Our response:** As suggested by reviewer, we understand the importance of animal model for the assessing the clinical applicability. The animal model commonly used the immunodeficiency animal model to assess the activity of the inhibitors, but the model is under the lack of host immunity-tumor cell interaction that may not truly reflect the activity of inhibitors in the in vivo assay. Furthermore, the application of radiotherapy in animal model is not easy to perform. Therefore, we are searching the best solution for the animal model in the further study.

4) Supporting genetic approaches to model changes to PI3K isoforms and their regulation of the DNA damage response.
**Our response:** As suggested by reviewer, the expression of γH2AX and PARP, which represent the DNA damage response, by western blotting will be checked in the combination treatment of PI3K inhibitor and radiotherapy in HNSCC animal model in the further study.

5) Evaluation of alternative modes of DNA damage to determine if the phenotype is unique to radiation and/or DNA double strand break induction.

**Our response:** The alternative modes of DNA damage will be identified by performing NGS assay to comprehensively investigate the mechanism in the combination treatment of PI3K inhibitor and radiotherapy in HNSCC animal model in the further study.

Reviewer #2: This study describes the effects of the PI3Kα inhibitors Alpelisib (BYL719) and Buparlisib (BKM120) on the radiation sensitivity of head and neck cancer cells. Although these studies are of some interest, they are limited in scope and descriptive. There are no mechanistic studies. In addition, the lack of in vivo studies makes it difficult to know if these agents and the combination with the mTOR inhibitor will translate into an actual treatment in patients.


1) Do the authors know if the concentrations used in the in vitro experiments are clinically relevant? What concentrations are found in patients receiving these drugs?

**Our response:** Yes, we know the relevant between the in vitro experiments and clinical usage dose. The concentration of alpelisib used in patients is at a dose of 300 mg per day, and the concentration of buparlisib used in patients is 100 mg per day.

2) The use of a single radiation dose (Figures 2 and 3) give limited information on overall radiation sensitivity. This is usually best assessed by performing a full cell survival curve of control and 3-4 radiation doses.

**Our response:** The suggestion by reviewers was ideal to support more information. Our unshown results demonstrated that different radiation doses dose-dependent reduced the cell growth when treated alone or combination treatment with PI3K inhibitors. We added the result into the manuscript as figure 3.

3) Did the agents actually inhibit PI3Kα under the conditions that were investigated for radiosensitization? This would be the best way to verify that the effects.

**Our response:** The suggestion by reviewers was ideal to confirm the effect of PI3K inhibitor. The pan-PI3K inhibitor buparlisib (BKM120) and the PI3K inhibitor alpelisib (BYL719) are developed by Novartis Oncology for clinical patients, and the specific of the inhibitors against PI3K is definite under the conditions.

4) Why did the authors choose combine these inhibitors with an mTOR inhibitor.

**Our response:** The PI3K/AKT/mTOR signaling pathway is a common mechanism to initiate the survival and proliferation. Therefore, the combination treatment of PI3K and mTOR inhibitor may boost the anticancer effect in the OSCC cells.

5) The MTT like assay is useful for screening large number of conditions rapidly but is not as accurate as a clonogenic assay for detailed assessment of radiosensitization

**Our response:** As suggested by reviewer, the MTT assay in present study was the primary experiments to determine the available concentrations of the inhibitors for the further assay. Using clonogenic assay to investigate the appropriate concentrations is more time consuming.

6) The clinical applicability of these combinations is difficult to assess with in vitro experiments alone.

**Our response:** As suggested by reviewer, we understand the importance of animal model for the assessing the clinical applicability. The animal model commonly used the immunodeficiency animal model to assess the activity of the inhibitors, but the model is under the lack of host immunity-tumor cell interaction that may not truly reflect the activity of inhibitors in the in vivo assay. Furthermore, the application of radiotherapy in animal model is not easy to perform. Therefore, we are searching the best solution for the animal model in the further study.
