# Supplementary material for: PI3k inhibitors (BKM120 and BYL719) as radiosensitizers for head and neck squamous cell carcinoma during radiotherapy
Source: PLoS One. 2021 Jan 20;16(1):e0245715. doi: 10.1371/journal.pone.0245715 (PMC7817006; doi:10.1371/journal.pone.0245715)
Supplement: Attachment — Submitted filename: 20201207_Response to reviewer.docx [file pone.0245715.s002.docx]

Review Comments to the Author

Please use the space provided to explain your answers to the questions above. You may also include additional comments for the author, including concerns about dual publication, research ethics, or publication ethics. (Please upload your review as an attachment if it exceeds 20,000 characters)

**Reviewer #2:**

The authors have responded to some comments but not some
critical ones.
Comment 1) - the question was about concentrations and the authors
have answered in mg

**Our response:** As suggested by reviewer, the concentrations of the inhibitors in patients is associated with how many mg of the inhibitor that patient took, and the inhibitors concentration in the patient plasma appear dynamic which was showed as pharmacokinetics. For example, the alpelisib used in patients at a dose of 350 mg per day in cycle 1 showed the median maximum plasma concentration (Cmax) is 3490 ng/ml. The buparlisib used in patients at a dose of 100 mg per day in cycle 1 showed the Cmax is 1080 ng/ml.

Comments 5 and 6). this reviewer agrees that performing clonogenic
assays and animal experiments is time consuming. But they provide
important information concerning the clinical applicability of this
combination.

**Our response:** As suggested by reviewer, we understand and agree that the animal model is important information for clinical applicability of the combination. However, previous studies had indicated that the PI3K/Akt/mTOR pathway inhibitors plus radiotherapy were ideal strategy for lung cancer and breast cancer (1, 2). Furthermore, several PI3K/Akt/mTOR inhibitors including alpelisib and buparlisib has been involved in clinical trials, indicating that the use of the inhibitor is available (3, 4). The combination treatment of PI3K/Akt/mTOR inhibitor and radiotherapy has also been identify that the strategy is clinical applicable(1). On the other hand, the use of the hospital facility was limited because of the novel coronavirus disease, which has influenced the progress of the study. In the near future, we will proceed the in vivo analysis to provide the direct evidence.

Reference:

1.	Chen K, Shang Z, Dai AL, Dai PL. Novel PI3K/Akt/mTOR pathway inhibitors plus radiotherapy: Strategy for non-small cell lung cancer with mutant RAS gene. Life sciences. 2020;255:117816.

2.	DuRoss AN, Neufeld MJ, Landry MR, Rosch JG, Eaton CT, Sahay G, et al. Micellar Formulation of Talazoparib and Buparlisib for Enhanced DNA Damage in Breast Cancer Chemoradiotherapy. ACS applied materials & interfaces. 2019;11(13):12342-56.

3.	Rodon J, Brana I, Siu LL, De Jonge MJ, Homji N, Mills D, et al. Phase I dose-escalation and -expansion study of buparlisib (BKM120), an oral pan-Class I PI3K inhibitor, in patients with advanced solid tumors. Investigational new drugs. 2014;32(4):670-81.

4.	Beck JT, Ismail A, Tolomeo C. Targeting the phosphatidylinositol 3-kinase (PI3K)/AKT/mammalian target of rapamycin (mTOR) pathway: an emerging treatment strategy for squamous cell lung carcinoma. Cancer treatment reviews. 2014;40(8):980-9.

**Reviewer #3:**

This study aimed to identify PI3K inhibitors that can enhance radiosensitivity in head and neck cancer cells. The authors showed that a combination of BKM120 or BYL719 with irradiation leads to an increase in radiosensitivity in HNSCC cells. After a meticulous review of the manuscript, I found that the current study could be pertinent and informative for the readers this journal. However, I think that there is some room for improvements before a further consideration for publication. At this point there is a need for minor revision before the manuscript could be reconsidered for publication in the journal. According to my assessments, there are some points to clarify and revise: In addition to radiotherapy, surgery is a widely used therapeutic option in patients with HNSCC. This should not remain unmentioned in the discussion, since surgery is superior to radiation therapy in early stages. Furthermore, in advanced tumors, radiotherapy is used in combination with hemotherapy and not as the sole modality. Theauthors should take a more differentiated view on the therapy of HNSCC and side effects of radiotherapy in the discussion section. I highly recommend investigating the effects of BKM120 and BYL719 in vivo in the near future.
**Our response:** As suggested by reviewer, we added more discussion about the importance of surgery and the side effect when patients treated with radiotherapy or chemotherapy in the discussion section. We will proceed the in vivo analysis soon. Because of the novel coronavirus disease, the use of the hospital facility was limited that also influence the progress of the study.
